# Effects of total knee arthroplasty on symptoms, function and activity over 5 years in knee osteoarthritis: A propensity‐score matched study

**DOI:** 10.1002/jeo2.70185

**Published:** 2025-03-22

**Authors:** Liru Ge, Junjie Wang, Haonan Fang, Yining Wang, Ziyuan Shen, Guoqi Cai

**Affiliations:** ^1^ Department of Epidemiology and Biostatistics, School of Public Health Anhui Medical University Hefei Anhui China; ^2^ Menzies Institute for Medical Research University of Tasmania Hobart Tasmania Australia

**Keywords:** osteoarthritis, pain, physical activity, physical function, total knee replacement

## Abstract

**Purpose:**

To evaluate the effects of total knee arthroplasty (TKA) on symptoms, function and activity over 5 years in knee osteoarthritis (KOA) patients.

**Methods:**

Data were from the Osteoarthritis Initiative (OAI). Participants who conducted the first TKA from (not before) enrolment to 48 months were propensity score matching (PSM) on their characteristics at the visit before surgery (treated as baseline) to those who did not conduct a TKA at 48 months (treated as baseline). Changes in knee pain and functional disability were evaluated using the Western Ontario and McMaster Universities Osteoarthritis Index (WOMAC) pain and function subscales. Changes in physical activity, physical function and overall physical health and mental health were assessed using the Physical Activity Scale for the Elderly, the 20‐m walk speed and the five times chair‐to‐stand tests and the 12‐Item Short Form Survey, respectively.

**Results:**

Eighty‐two pairs of participants in the TKA (56% women, mean 64.8 ± 8.4 years) and non‐TKA groups were matched. Knee symptoms were significantly improved in the TKA group and flatted from 24 months (Pain: *β* = −3.29, 95% confidence interval [CI] = [−4.59 to −1.99], *p* < 0.001; Function: *β* = −10.12, 95% CI = [−14.21 to −6.03], *p* < 0.001). Physical function and overall physical health but not physical activity or mental health (PASE: *β* = 5.72, 95% CI = [−15.46 to 26.90], *p* = 0.597; Mental: *β* = 0.04, 95% CI = [−2.47 to 2.54], *p* = 0.976) was improved in the TKA group over 24 months.

**Conclusions:**

TKA substantially improved knee symptoms and physical function over 60 months and physical health over 48 months, compared to those who had a similar severity of KOA but did not have a TKA, but this did not translate into increased physical activity or mental health.

**Level of Evidence:**

Level III.

## Graphical abstract

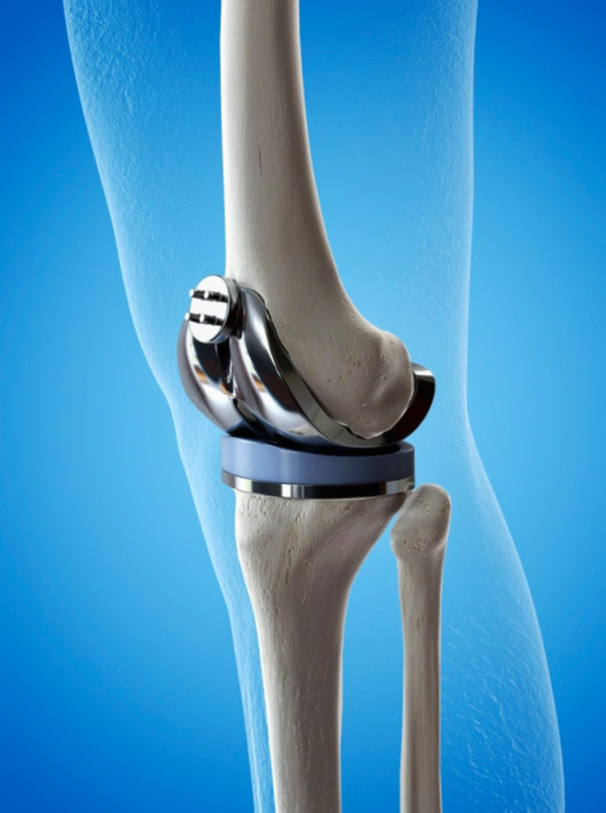

**Abbreviations**
20MWS: 20‐m walk speed
5CST: five times chair‐to‐stand test
5STS: five times sit‐to‐stand test
KL: Kellgren–Lawrence
KOA: knee osteoarthritis
KOOS: Knee Injury and Osteoarthritis Outcome Score
MCS: Mental Component Score
OAI: Osteoarthritis Initiative
PASE: Physical Activity Scale for the Elderly
PCS: Physical Component Score
PS: propensity score
PSM: propensity score matching
SF‐12: 12‐item Short Form Health Survey
SMD: standardized mean difference
TKA: total knee arthroplasty
WOMAC: Western Ontario and McMaster Universities Osteoarthritis Index

## INTRODUCTION

Knee osteoarthritis (KOA) is one of the most common joint diseases in the world and a leading cause of disability [34]. The rising prevalence of obesity and the aging population worldwide have both contributed to an increased burden of KOA [40]. Knee pain and functional disability due to KOA can significantly reduce physical function and limit physical activity levels, thus increasing the risk of other conditions such as obesity, diabetes and cardiovascular diseases [23, 26, 53]. For patients with advanced KOA whose knee symptoms do not respond to general interventions, total knee arthroplasty (TKA) is the only treatment option. While TKA effectively alleviates symptoms and enhances knee function [10, 16, 37, 50], studies have shown that the level of physical activity does not usually improve significantly after surgery [12, 33]. More importantly, studies have shown that approximately 7%–20% of patients express dissatisfaction with their post‐operative results [13, 15, 21]. There is a diversity of reasons for post‐operative dissatisfaction. Previous studies have noted that preoperative anxiety, depression and high expectations may negatively affect patients undergoing TKA, influencing their subjective function and pain perception before and 2 years after surgery [1, 27, 41]. In addition, studies have shown that persistent or recurring post‐operative pain, functional limitations and post‐operative lifestyle changes have also been identified as one of the main causes of post‐operative dissatisfaction [6, 7, 10, 22]. Previous studies mainly focused on comparing preoperative and post‐operative symptoms in patients receiving TKA [5, 31, 32]; few have evaluated whether TKA was superior to nonsurgical treatments for knee symptoms and related physical function and physical activity. In the randomized controlled trial by Skou et al. [46], patients with moderate‐to‐severe KOA were enrolled and divided into two groups for comparison. One group underwent TKA followed by a 12‐week nonsurgical treatment regimen, which included interventions such as exercise, education, dietary advice, use of insoles and pain medication. The second group received only the 12‐week nonsurgical treatment. This study demonstrated that patients in the TKA group experienced greater improvements in knee symptoms, physical function and quality of life after 12 months compared to those in the nonsurgical group. This study demonstrated that patients in the total knee replacement group experienced greater improvements in knee symptoms, physical function, and quality of life after 12 months compared to those in the nonsurgical treatment group. Subsequent analyses showed that symptomatic relief persisted in the TKA‐treated group after 24 months [47]. Moreover, it is also important to know whether the improved knee symptoms can be translated into increased physical function and activity, thereby reducing the risk of other chronic diseases [9, 19].

This study used data from a cohort of older adults with or at increased risk of KOA to compare the effect of TKA versus non‐TKA on knee symptoms, physical function and physical activity.

## METHODS

### Data sources

Data were from the Osteoarthritis Initiative (OAI), an open multicenter, longitudinal, prospective observational study of adults with or at increased risk of symptomatic KOA (https://nda.nih.gov/oai). Individuals aged from 45 to 79 years were recruited from four clinical sites in the United States. Major exclusion criteria included inflammatory arthritis (e.g., rheumatoid arthritis), bilateral end‐stage KOA, bilateral TKA and contraindications to 3‐T magnetic resonance imaging. All participants were scheduled for a clinical visit every 12 months since 2002, with a maximum follow‐up of 108 months. Ethical approval was obtained from the institutional review boards of all four clinical centres participating in OAI. All participants provided written informed consent.

### Study design

**Figure 1:**
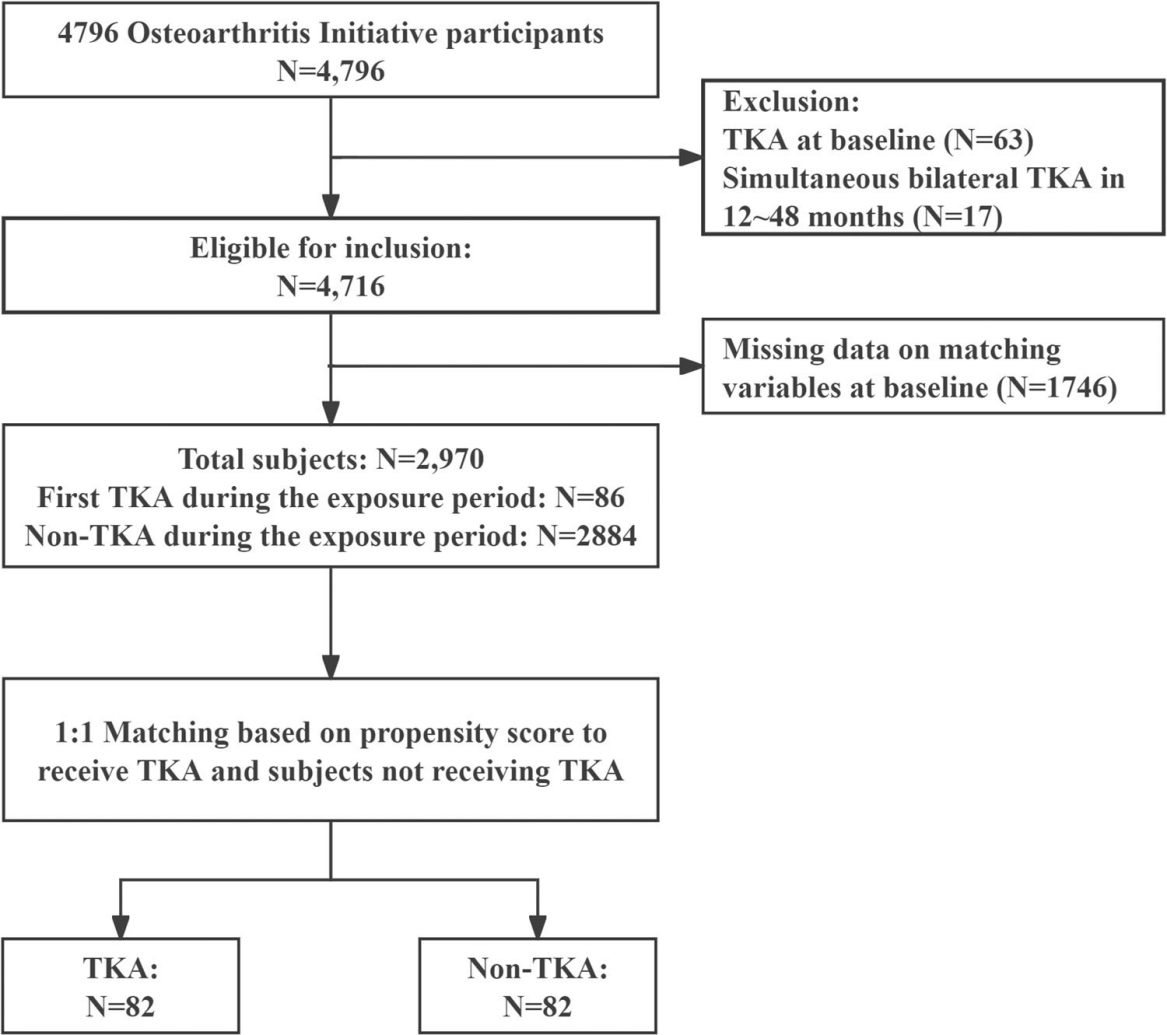
Subject selection from OAI database. n, number of participants included in the analysis; OAI, Osteoarthritis Initiative; TKA, total knee arthroplasty.

**Figure 2:**
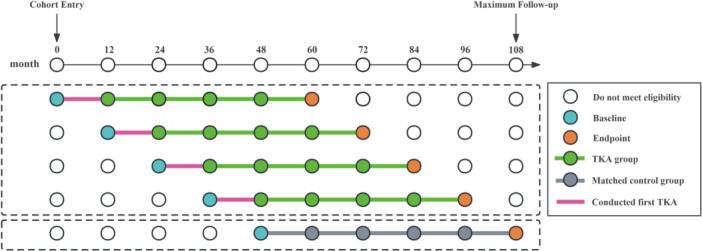
Demonstrate matching of TKA and non‐TKA groups in the OAI cohort. Participants in the TKA group had their first TKA from the cohort entry (not before) to 48 months, and the baseline for this group was the clinical visit before the TKA was performed. The propensity score matching control group was selected from participants who did not conduct a TKA at or prior to the 48‐month visit. OAI, Osteoarthritis Initiative; TKA, total knee arthroplasty.

In this study, we excluded participants who had had a TKA before cohort entry because the aim was to evaluate changes in knee symptoms and physical function and activity before and after TKA. We also excluded participants who had undergone simultaneous bilateral TKA because the development of knee symptoms may differ with unilateral TKA [11] (see flowchart in Figure 1). To ensure an adequate sample size of participants who had TKA as well as a long follow‐up period for the evaluation of the effect of TKA on the outcome measures, we prespecified the exposure period from cohort entry to the 48 months (the landmark date) [38]. Participants who had had their first TKA during the exposure period were included in the TKA group, for whom the latest annual visit before the TKA was considered baseline (Figure 2). Participants who did not have any TKA until month 48 were included in the non‐TKA group, for whom the 48‐month visit was considered baseline (Figure 2). For both the TKA and non‐TKA groups, we extracted data on participants for a maximum of 60 months.

### Primary outcomes

The Western Ontario and McMaster Universities Osteoarthritis Index (WOMAC) pain and function scores were used to assess joint pain (0–20) and functional disability (0–68) in the left and right knees, with higher scores indicating more severe symptoms [8]. This was done every 12 months over 60 months.

### Secondary outcomes

The Physical Activity Scale for the Elderly (PASE) was used to measure a variety of physical activities during daily life, including household chores, work, leisure activities and exercises and walking and bicycling [52]. PASE uses a scale of 0–400, with higher scores representing higher levels of physical activity [48].

Physical ability was assessed using the 20‐m walk speed (20MWS) and the five times chair‐to‐stand tests (5CST). For the 20MWS, participants were told to walk at a normal walking speed from start to finish, and the speed was measured in metres per second. For the 5CST, participants stood from a chair, keeping their arms crossed over their chest. Participants were told that the required manoeuvre was to go from a seated position with arms crossed in front of the chest and hips touching the chair to a full standing position and then sitting down again, which was recorded as a chair‐to‐stand activity [2]. These two metrics were used to assess the recovery of lower extremity strength and function of patients.

The Physical Component Score (PCS) and Mental Component Score (MCS) from the 12‐item Short Form Health Survey (SF‐12) questionnaire were used to assess the overall physical and mental health status, respectively [14]. The physical indicators in the SF‐12 scale provide a broader assessment of an individual's overall physical health than the WOMAC function indicators [8, 51].

These secondary outcomes were assessed every 24 months from the landmark date in the OAI; therefore, we evaluated change in these outcomes over 24 and 48 months.

### Covariates

The selection of covariates was based on previous studies for their potential confounding effects on the symptomatic progression of KOA [28]. They were measured or recorded at baseline, including age (years) [24], sex (man/woman) [17], body mass index (BMI; kg/m^2^) [44], educational level, annual household income, race (White and Other) [45] and the Kellgren–Lawrence (KL) scores for both knees using x‐ray.

### Data analyses

Descriptive statistics were employed to summarize the baseline characteristics of the study population. For categorical measures, the frequency and percentage (%) of knees in each group were reported. As for continuous variables, the means and standard deviations (mean ± SD) were provided. Statistical tests such as ANOVA and chi‐square were utilized to compare the groups and assess any significant differences. Using logistic regression modelling, we determined the propensity score (PS) to undergo TKA during the exposure period. Covariates entered the propensity score were sociodemographic (age, sex, BMI, education, annual household income and race), KOA severity (KL score in both knees, WOMAC pain and WOMAC function scores), and health status (SF‐12, PASE, 20MWS and 5CST) at baseline. We used callipers with a width equal to the log standard deviation of the propensity score of 0.2. The nearest‐neighbour matching method was used to match each individual in the TKA group to one individual in the non‐TKA group based on the proximity of the PS between individuals in the two groups [4].

The standardized mean difference (SMD) was measured to determine the covariate balance before propensity score matching (PSM) and after PSM with SMD > 0.1 indicating an imbalance. We developed a GEE using a Gaussian distribution and homogeneity link function to test the effect of TKA on the outcome measures, using an exchangeable correlation structure to account for within‐subject correlations. The ‘treatment’ and ‘month’ variables and their interaction were included as predictors, with adjustment for imbalanced covariates post‐PSM (i.e., SMD > 0.1). Multiple imputation by chained equations was used to address missing data caused by loss to follow‐up and nonresponses, with five imputations performed assuming missing at random.

Complete‐case analysis was performed as a sensitivity analysis. Another sensitivity analysis was conducted by excluding participants in the TKA group (and matched controls) who underwent a contralateral TKA surgery and follow‐up data on participants in the non‐TKA group from the implementation of a TKA.

All statistical analyses were performed by R software (version 4.2.1). Statistical significance was set at a *p* value of less than 0.05 (two‐tailed).

## RESULT

### Cohort characteristics

Out of the initial 4796 cohort participants, 63 had undergone TKA before cohort entry, and 17 underwent simultaneous bilateral knee replacements during the exposure period. Due to missing variables for inclusion matching at baseline, 1746 participants were excluded from the matching process. Subsequently, 86 participants experienced their first TKA during the exposure period. Following 1:1 PSM with patients who had not undergone TKA during this period, 82 pairs of participants (mean 65.24 years, 57.9% female) were selected for inclusion in our study (Figure 1).

### Matching

Table 1 shows the baseline characteristics of participants in the TKA and non‐TKA groups before and after PSM. Eighty‐two participants (56% women, mean 64.8 ± 8.4 years) in the TKA group were 1:1 matched to 82 participants in the non‐TKA group. No statistically significant differences were found in the variables included for matching the TKA and non‐TKA groups after PSM.

**Table 1:** Baseline characteristics of study participants before and after propensity score matching (PSM).

	Before PSM				After PSM			
	No‐TKA (*N* = 2868)	TKA (*N* = 86)	*p*	SMD	No‐TKA (*N* = 82)	TKA (*N* = 82)	*p*	SMD
Age (mean (SD))	64.28 (8.92)	64.64 (9.06)	0.710	0.041	65.71 (8.39)	64.77 (8.98)	0.490	0.108
Sex (%)								
Male	1283 (44.49)	36 (41.86)	0.709	0.053	33 (40.24)	36 (43.90)	0.752	0.074
Female	1601 (55.51)	50 (58.14)			49 (59.76)	46 (56.10)		
BMI (mean (SD))	28.471 (4.84)	30.45 (4.69)	<0.001	0.415	30.75 (5.23)	30.25 (4.65)	0.520	0.101
Education level (%)								
≤High school	365 (12.66)	20 (23.26)	0.008	0.312	20 (24.39)	19 (23.17)	0.361	0.224
College	1298 (45.01)	39 (45.35)			43 (52.44)	36 (43.90)		
Graduate degree	1221 (42.34)	27 (31.40)			19 (23.17)	27 (32.93)		
Income (%)								
<25k	306 (10.61)	7 (8.14)	0.907	0.086	10 (12.20)	7 (8.54)	0.852	0.139
25–50k	710 (24.62)	22 (25.58)			23 (28.05)	22 (26.83)		
50–100k	1093 (37.90)	33 (38.37)			30 (36.59)	31 (37.80)		
>100k	775 (26.87)	24 (27.91)			19 (23.17)	22 (26.83)		
Race (%)								
White	2393 (82.98)	73 (84.88)	0.750	0.052	68 (82.93)	69 (84.15)	1.000	0.033
Other	491 (17.02)	13 (15.12)			14 (17.07)	13 (15.85)		
KL in left knee (%)								
≤2	2365 (82.00)	30 (34.88)	<0.001	1.136	31 (37.80)	29 (35.37)	0.934	0.058
3	370 (12.83)	24 (27.91)			23 (28.05)	23 (28.05)		
4	149 (5.17)	32 (37.21)			28 (34.15)	30 (36.59)		
KL in right knee (%)								
≤2	2342 (81.21)	27 (31.40)	<0.001	1.185	21 (25.61)	26 (31.71)	0.656	0.144
3	407 (14.11)	33 (38.37)			32 (39.02)	31 (37.80)		
4	135 (4.68)	26 (30.23)			29 (35.37)	25 (30.49)		
WOMAC Pain in left knee (mean (SD))	1.71 (2.85)	5.00 (4.30)	<0.001	0.902	4.60 (4.45)	4.76 (4.18)	0.815	0.037
WOMAC Pain in right knee (mean (SD))	1.90 (2.83)	5.05 (3.87)	<0.001	0.927	5.65 (4.19)	4.90 (3.79)	0.235	0.186
WOMAC Function in left knee (mean (SD))	5.66 (9.42)	18.11 (13.38)	<0.001	1.076	16.70 (13.93)	17.26 (12.94)	0.791	0.042
WOMAC Function in right knee (mean (SD))	5.93 (9.30)	16.44 (13.61)	<0.001	0.903	16.67 (12.39)	15.83 (13.34)	0.677	0.065
SF‐12 Mental (mean (SD))	54.09 (8.19)	56.60 (6.99)	0.005	0.329	55.39 (9.04)	56.24 (6.95)	0.500	0.106
SF‐12 Physical (mean (SD))	48.99 (8.77)	38.71 (9.85)	<0.001	1.103	38.84 (10.55)	39.34 (9.48)	0.750	0.050
PASE (mean (SD))	159.56 (81.88)	133.49 (79.93)	0.004	0.322	139.26 (77.26)	136.62 (80.41)	0.831	0.033
20‐m speed (mean (SD))	1.33 (0.20)	1.19 (0.22)	<0.001	0.650	1.18 (0.21)	1.20 (0.22)	0.548	0.094
The 5‐time chair‐to‐stand tests (mean (SD))	10.86 (3.43)	13.43 (4.30)	<0.001	0.661	12.40 (3.30)	13.54 (4.28)	0.058	0.299

Abbreviations: BMI, body mass index; KL, Kellgren–Lawrence; PASE, Physical Activity Scale for the Elderly; SD, standard deviation; SF‐12, Short Form 12‐Item Health Survey; SMD, standardized mean difference; TKA, total knee arthroplasty; WOMAC, Western Ontario and McMaster Universities arthritis index.

### Primary outcome

**Figure 3:**
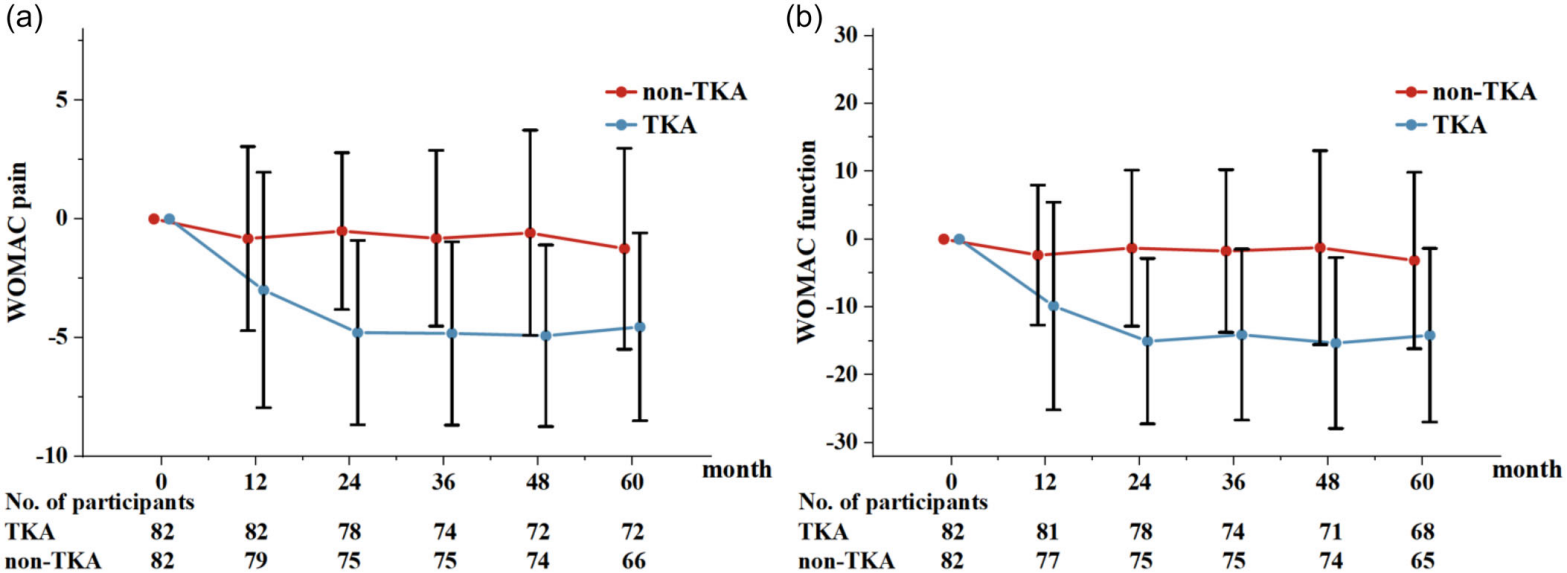
Change in (a) the Western Ontario and McMaster University Index (WOMAC) pain and (b) function scores from baseline in the TKA and non‐TKA groups over 60 months. *Positive scores indicate worsening and negative improvement from baseline. Vertical bars indicate 95% CIs for the mean scores. CI, confidence interval; TKA, total knee arthroplasty.

The treatment effect of TKA on knee symptoms continued to increase until 24 months and plateaued thereafter (Pain: *β* = −2.09 to −3.29, *p* < 0.001; Function: *β* = −7.05 to −10.12, *p* < 0.001) (Figure 3). In contrast, knee symptoms in the non‐TKA group were stable throughout the study. From baseline to the 60‐month follow‐up, both knee pain and functional disability in the TKA group showed a greater improvement compared to the non‐TKA group (Table 2).

**Table 2:** Change in knee symptoms between the TKA and non‐TKA groups over 60 months.

	TKA		Non‐TKA		Between‐group difference TKA − Non‐TKA	
	*β* (95% CI)	*p*	*β* (95% CI)	*p*	*β* (95% CI)	*p*
WOMAC Pain (0–20)						
12	−3.00 (−4.07 to −1.93)	<0.001	−0.91 (−1.87 to 0.04)	0.062	−2.09 (−3.46 to −0.72)	0.003
24	−4.73 (−5.69 to −3.77)	<0.001	−0.39 (−1.54 to 0.76)	0.510	−4.26 (−5.38 to −3.14)	<0.001
36	−4.47 (−5.69 to −3.24)	<0.001	−0.81 (−1.99 to 0.36)	0.182	−3.83 (−5.01 to −2.65)	<0.001
48	−4.46 (−5.87 to −3.06)	<0.001	−0.45 (−1.83 to 0.92)	0.522	−4.24 (−5.49 to −2.98)	<0.001
60	−4.09 (−5.33 to −2.85)	<0.001	−0.72 (−2.77 to 1.33)	0.499	−3.29 (−4.59 to −1.99)	<0.001
WOMAC Function (0–68)						
12	−9.84 (−13.19 to −6.49)	<0.001	−2.05 (−5.13 to 1.03)	0.197	−7.05 (−11.09 to −3.01)	<0.001
24	−14.40 (−17.51 to −11.29)	<0.001	−0.46 (−4.41 to 3.49)	0.819	−12.99 (−16.70 to −9.28)	<0.001
36	−13.42 (−17.12 to −9.72)	<0.001	−0.59 (−5.29 to 4.12)	0.809	−11.85 (−15.69 to −8.01)	<0.001
48	−13.90 (−17.98 to −9.82)	<0.001	−0.01 (−5.31 to 5.29)	0.998	−12.96 (−17.19 to −8.73)	<0.001
60	−12.81 (−17.55 to −8.07)	<0.001	0.06 (−8.76 to 8.88)	0.990	−10.12 (−14.21 to −6.03)	<0.001

*Note*: Adjust for covariates at baselines: age, BMI, education, income, KL score of the right knee, Pain scores in the right knee, mental score of SF‐12 and the five times chair‐to‐stand tests.

Abbreviations: 95% CI, 95% confidence interval; BMI, body mass index; KL, Kellgren–Lawrence; SF‐12, Short Form 12‐Item Health Survey; TKA, total knee arthroplasty; WOMAC, Western Ontario and McMaster University Osteoarthritis.

### Secondary outcomes

**Figure 4:**
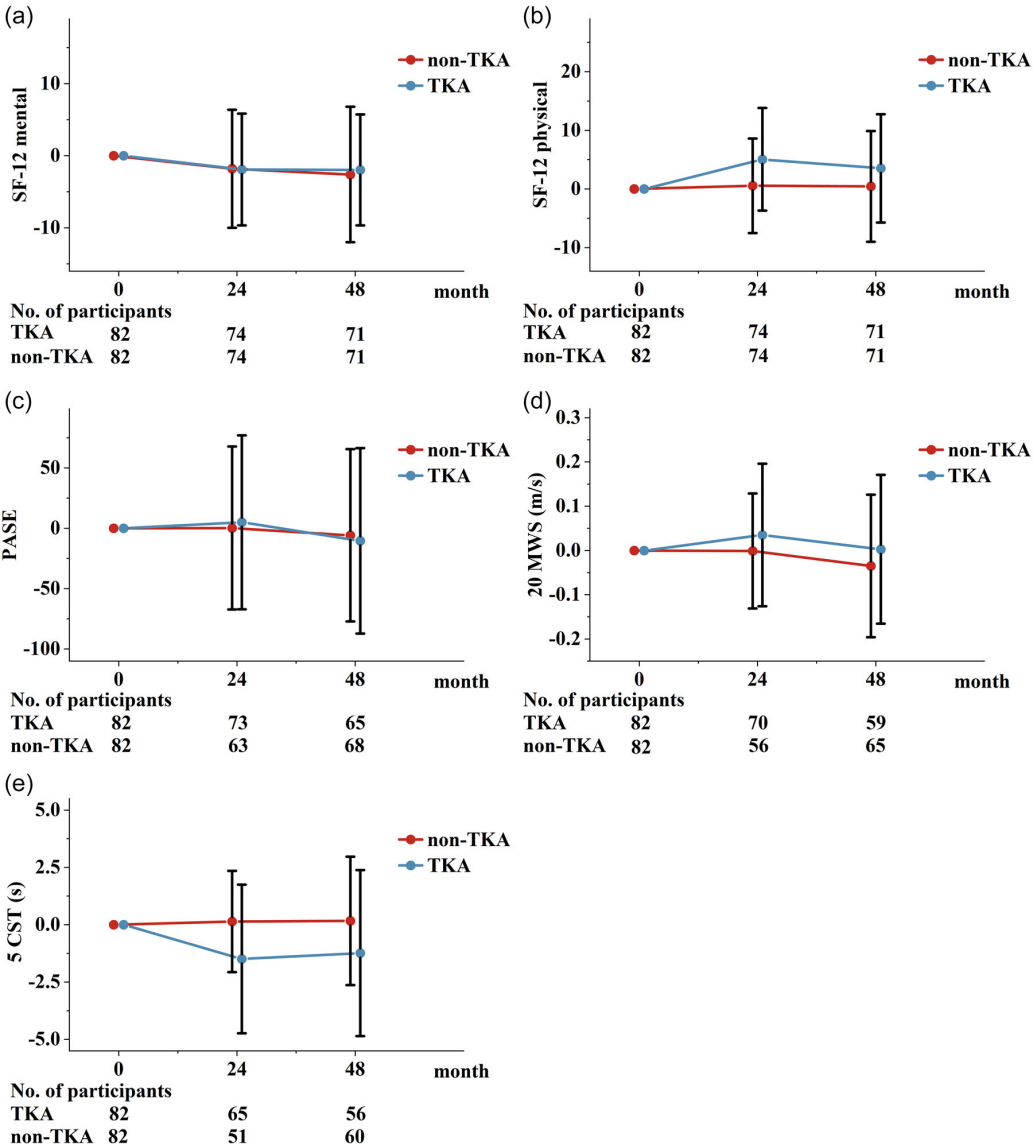
Comparison between the TKA and non‐TKA groups on change in secondary outcome. (a) The change of SF‐12 Mental, Positive scores indicate improvement and negative indicate deterioration from baseline. (b) The change of SF‐12 Physical, Positive scores indicate improvement and negative indicate deterioration from baseline. (c) The change of PASE, Positive scores indicate improvement and negative indicate deterioration from baseline. (d) The change of 20‐m walk speed, Positive scores indicate improvement and negative indicate deterioration from baseline. (e) the change of five times chair stands, timed, Positive scores indicate worsening and negative improvement from baseline. Vertical bars indicate 95% CIs for the mean scores. 95% CI, 95% confidence interval; PASE, Physical Activity Scale for the Elderly; SF‐12, Short Form 12‐Item Health Survey; TKA, total knee arthroplasty.

Physical function (20MWS and 5CST) and overall physical health (SF‐12 physical health) were improved in the TKA group at 24 months and slightly regressed at 48 months (Physical: *β* = 4.18, 95% CI = [1.45–6.91], *p* = 0.003; 20MWS: *β* = 0.04, 95% CI = [−0.01 to 0.09], *p* = 0.093; 5CST: *β* = −1.41, 95% CI = [−2.34 to −0.48], *p* = 0.003) (Figure 4). Mental health score (SF‐12 mental health) and physical activity (PASE) were slightly decreased in both groups (PASE: β = 5.72, 95% CI = [−15.46 to 26.90], *p* = 0.597; Mental: *β* = 0.04, 95% CI = [−2.47 to 2.54], *p* = 0.976). For between‐group comparisons (Table 3), overall physical health and 5CST were significantly improved over 24 and 48 months in the TKA group, compared to the non‐TKA group, and 20MWS showed a statistically non‐significant trend to be improved in the TKA group. There were no significant between‐group differences for changes in mental health score and physical activity over 24 or 48 months (Table 3).

**Table 3:** Change in physical/mental health, physical function and physical activity between the TKA and non‐TKA groups over 48 months.

	TKA		Non‐TKA		Between‐group difference TKA − Non‐TKA	
	*β* (95% CI)	*p*	*β* (95% CI)	*p*	*β* (95% CI)	*p*
SF‐12 Mental (0‐100)						
24	−1.48 (−4.20 to 1.25)	0.298	−1.93 (−5.72 to 1.86)	0.334	0.04 (−2.47 to 2.54)	0.976
48	−2.22 (−5.66 to 1.21)	0.223	−2.86 (−7.08 to 1.37)	0.205	0.73 (−1.92 to 3.38)	0.589
SF‐12 Physical (0‐100)						
24	5.62 (2.18–9.06)	0.005	−0.08 (−3.95 to 3.78)	0.967	4.18 (1.45–6.91)	0.003
48	4.85 (0.37–9.33)	0.052	−0.11 (−5.20 to 4.98)	0.967	2.90 (−0.07 to 5.87)	0.057
PASE (0‐400)						
24	7.50 (−29.39 to 44.39)	0.696	1.88 (−61.17 to 64.92)	0.955	5.72 (−15.46 to 26.90)	0.597
48	−0.87 (−60.74 to 59.00)	0.978	−6.25 (−53.44 to 40.93)	0.801	−1.73 (−24.13 to 20.66)	0.880
20‐m speed (m/s)						
24	0.06 (−0.05 to 0.17)	0.337	0.01 (−0.21 to 0.23)	0.908	0.04 (−0.01 to 0.09)	0.093
48	0.05 (−0.16 to 0.26)	0.672	0.00 (−0.16 to 0.15)	0.971	0.04 (−0.01 to 0.09)	0.114
The 5‐time chair‐to‐stand tests (s)						
24	−1.00 (−3.88 to 1.88)	0.512	−0.22 (−4.58 to 4.15)	0.927	−1.41 (−2.34 to −0.48)	0.003
48	−0.56 (−4.70 to 3.58)	0.797	−0.20 (−3.12 to 2.73)	0.900	−1.23 (−2.43 to −0.03)	0.048

*Note*: Adjust for covariates at baselines: age, BMI, education, income, KL score of the right knee, Pain scores in the right knee, mental score of SF‐12 and the five times chair‐to‐stand tests.

Abbreviations: 95% CI, 95% confidence interval; BMI, body mass index; KL, Kellgren–Lawrence; PASE, Physical Activity Scale for the Elderly; SF‐12, 12‐item Short Form Health Survey; TKA, total knee arthroplasty.

### Sensitivity analyses

Complete‐case analysis did not materially change the main findings (Table S1). During the 60‐month follow‐up, 8 participants in the TKA group underwent a contralateral TKA surgery and 17 in the non‐TKA group conducted a TKA. The exclusion of the 8 participants in the TKA group and matched controls and follow‐up data on the 17 participants in the non‐TKA group from the implementation of a TKA did not change the main findings, except that the between‐group effect of TKA on change in 20MWS over 48 months became statistically significant (Table S2).

## DISCUSSION

In this study, we found that compared to the non‐TKA group, patients in the TKA group had substantially greater improvement in knee pain and knee function, physical function and overall physical health throughout the study period. However, no significant between‐group differences were found in changes in mental health and physical activity. These findings suggest a strong and long‐term effect of TKA on knee symptoms and physical function/health, but the effect has not been translated into increased physical activity, which is crucial to other chronic diseases.

In a randomized controlled trial conducted by Skou et al., TKA followed by non‐surgical treatment was more effective in improving knee pain and function over 12 months compared to non‐surgical treatment alone [46]. Post hoc analyses also indicated a significant and stable effect of TKA on knee symptoms and quality of life over 24 months [47]. Our study confirmed these findings and further indicated an effect of TKA on knee symptoms over 60 months, and the effect was not attenuated until the end of follow‐up. An explanation for these findings could be that, in the first 2 years post‐surgery, patients typically participate in various post‐operative rehabilitation protocols, including physiotherapy, rehabilitation exercises and regular follow‐up [20, 25, 36]. Such measures may alleviate post‐operative pain and facilitate joint function recovery. Thus, the gradual reduction in pain may reflect the effectiveness of these rehabilitation strategies. After 2 years, patients may reach a stable rehabilitation phase, where pain levels may reach a manageable threshold, with further improvement potentially constrained by the limits of post‐operative rehabilitation. These findings support current opinions that TKA surgery is a safe and effective treatment approach for relieving knee symptoms [43, 49].

In our study, overall physical health and 5CST were significantly improved in the TKA group at both 24 and 48 months, although a trend of diminution was observed. Moreover, the between‐group difference in 20MWS was not statistically significant in the main analyses, and this can be due to the fact that the 20MWS test only reflects a normal walking speed, which is less influenced by knee symptoms. Our findings are mostly consistent with another study evaluating the impact of TKA on gait, the five times sit‐to‐stand test (5STS), and performance‐based and self‐reported functional outcomes within one year after surgery [29]. They observed significant improvements in gait patterns, 5STS and functional scores assessed using the Knee Injury and Osteoarthritis Outcome Score (KOOS) over 1 year, although TKA‐treated patients did not reach the levels observed in the healthy control group [29]. Taken together, TKA may improve the physical function of patients with KOA, but full restoration is unlikely.

In a systematic review [3], there were only marginal changes in PASE scores at 6 months post‐TKA, with limited evidence of more substantial changes at 1 year after surgery. Our study did not demonstrate significant increases in physical activity in the TKA group at 24 and 48 months, nor were noticeable improvements in psychological well‐being observed between 24 and 48 months. Various factors may have contributed to the lack of treatment effects on mental health improvement and physical activity levels post‐operatively. Inadequate post‐operative pain management, socioeconomic disparities affecting access to rehabilitation resources, and the perceived level of social support in the post‐operative period are all potential influencers of patients' motivation to effectively engage in physical activities and manage their mental well‐being. Moreover, fear of re‐injury, the presence of comorbidities, cultural beliefs around physical activity and mental health, and the absence of individualized rehabilitation plans tailored to patients' specific needs may further contribute to the discrepancy between improved physical function and stagnant mental health scores and physical activity levels post‐operatively. It is worth noting that this inconsistency may be attributed to the rehabilitation phase commonly experienced by patients [18], during which those with greater adherence to rehabilitation plans tend to achieve more favourable physical and mental recovery outcomes [35]. Over time, however, rehabilitation progress may slow or plateau, potentially leading to decreased physical activity levels.

Additionally, patients with preexisting mental health conditions may face heightened psychological pressures post‐surgery, potentially affecting their rehabilitation process and outcomes [39]. Although improvements in physical function may occur, they do not necessarily translate to increased levels of physical activity or benefits for other chronic conditions. Concerns about compromising the stability and longevity of the implant through high‐intensity physical activities may also deter patients from engaging in such activities. Moreover, patients with high preoperative expectations may experience dissatisfaction if post‐operative outcomes fall short of their expectations [30].

Scott et al. [42] indicated an elevated incidence of clinically significant depressive symptoms pre‐ and post‐operatively among TKA patients compared to the general population. A prolonged post‐operative recovery period may heighten the psychological burden, highlighting a need for further investigation into the mental health benefits following TKA. Insufficient physical activity not only compromises physical health but also adversely impacts mental health, further reducing patients' willingness to engage in physical activity. Therefore, the provision of a comprehensive rehabilitation programme that incorporates psychological counselling and structured rehabilitation guidance is essential for helping patients overcome these barriers.

The strengths of this study include the long follow‐up period and the matched non‐TKA group of patients who had a similar severity of KOA. Our study has several limitations. First, due to the lack of specific dates of TKA procedures in the OAI cohort, participants were asked only during the annual follow‐ups if they had undergone TKA surgery within the past year. To address this limitation, we selected the 48th month as a significant time point and included patients who underwent their first TKA surgery within the 0–48 months period in the exposed group. This approach allowed us to estimate TKA exposure reasonably while maintaining methodological consistency. Second, it is noteworthy that data collection for SF‐12, PASE, 20MWS and 5CST in the OAI cohort occurred every 2 years after the landmark date. Consequently, we considered these variables as secondary outcomes, using the two‐year interval as a designated time point for subsequent assessments. This approach allowed us to further investigate the effects of TKA beyond our primary outcome measures, thereby providing additional valuable insights into patient outcomes post‐surgery. Third, the sample size of this study was relatively small, which may have limited the generalizability and representativeness of the findings. As an observational study, we have maximized the use of available data and did not conduct power analysis due to the nature of the study design. However, further larger studies are warranted to confirm our findings regarding the lack of a significant effect of TKA on mental health and physical activity over 48 months. Finally, it is important to note that PSM, while effective in reducing selection bias, cannot account for unmeasured confounding variables inherent in observational studies. Factors such as patient preferences, socioeconomic status and unrecorded comorbidities could potentially influence the outcomes and were not captured in our analysis. Despite this, the effects of TKA on WOMAC pain and function over 12–24 months in our study were comparable to those from the 1‐year RCT and its 2‐year post‐hoc analysis by Skou et al., suggesting that our findings are robust.

In conclusion, TKA substantially improved knee symptoms, physical function and overall physical health post‐operation and remained stable over 48 or 60 months, compared to those who had a similar severity of KOA but did not have a TKA, but this did not translate into increased physical activity or mental health.

## AUTHOR CONTRIBUTIONS

**Liru Ge**: Methodology; investigation; data curation; writing—original draft; writing—review and editing. **Junjie Wang**: Conceptualization; investigation; formal analysis; writing—review and editing. **Haonan Fang**: Methodology; writing—review and editing. **Yining Wang**: Supervision, writing—review and editing. **Ziyuan Shen**: Supervision, writing—review and editing. **Rui Zhu**: Conceptualization; writing—review and editing. **Guoqi Cai**: Conceptualization; methodology; project administration; supervision; writing—review and editing.

## CONFLICT OF INTEREST STATEMENT

The authors declare no conflicts of interest.

## ETHICS STATEMENT

The ethics statement is not available.

## Supporting information

Supporting information.

## Data Availability

The data sets generated during and/or analyzed during the current study are available from the corresponding author on reasonable request.
