# Supplementary material for: Effects of total knee arthroplasty on symptoms, function and activity over 5 years in knee osteoarthritis: A propensity‐score matched study
Source: J Exp Orthop. 2025 Mar 22;12(1):e70185. doi: 10.1002/jeo2.70185 (PMC11928881; doi:10.1002/jeo2.70185)
Supplement: Supplementary file 1 — Supporting information. [file JEO2-12-e70185-s001.docx]

Supplementary Table 1. Complete-case analyses for the effect of TKA vs non-TKA

|  | **TKA** | | **non-TKA** | | **Between-Group Difference**  **TKA – non-TKA** | |
| --- | --- | --- | --- | --- | --- | --- |
|  | **beta (95% CI)** | **P** | **beta (95% CI)** | **P** | **beta (95% CI)** | **P** |
| **WOMAC Pain (0-20)** |  |  |  |  |  |  |
| 12 | -3.00 (-4.07 to -1.93) | <0.001 | -0.89 (-1.75 to -0.04) | 0.040 | -2.10 (-3.47 to -0.74) | 0.003 |
| 24 | -4.85 (-5.70 to -4.01) | <0.001 | -0.47 (-1.22 to 0.27) | 0.214 | -4.36 (-5.48 to -3.23) | <0.001 |
| 36 | -4.91 (-5.75 to -4.07) | <0.001 | -0.96 (-1.80 to -0.13) | 0.024 | -3.93 (-5.11 to -2.74) | <0.001 |
| 48 | -5.10 (-5.93 to -4.26) | <0.001 | -0.63 (-1.62 to 0.35) | 0.208 | -4.44 (-5.73 to -3.15) | <0.001 |
| 60 | -4.70 (-5.57 to -3.83) | <0.001 | -1.21 (-2.22 to -0.21) | 0.018 | -3.46 (-4.78 to -2.14) | <0.001 |
| **WOMAC Function (0-68)** |  |  |  |  |  |  |
| 12 | -9.87 (-13.16 to -6.57) | <0.001 | -2.64 (-4.92 to -0.37) | 0.023 | -7.19 (-11.21 to -3.18) | <0.001 |
| 24 | -14.86 (-17.50 to -12.21) | <0.001 | -1.38 (-3.93 to 1.17) | 0.287 | -13.41 (-17.09 to -9.73) | <0.001 |
| 36 | -14.31 (-17.08 to -11.54) | <0.001 | -2.09 (-4.78 to 0.59) | 0.126 | -12.15 (-16.01 to -8.29) | <0.001 |
| 48 | -15.19 (-17.99 to -12.40) | <0.001 | -1.43 (-4.62 to 1.76) | 0.380 | -13.71 (-17.95 to -9.47) | <0.001 |
| 60 | -14.13 (-16.96 to -11.30) | <0.001 | -2.94 (-6.05 to 0.18) | 0.065 | -11.05 (-15.25 to -6.84) | <0.001 |
| **SF-12 Mental (0-100)** |  |  |  |  |  |  |
| 24 | -1.80 (-3.56 to -0.04) | 0.045 | -1.71 (-3.60 to 0.17) | 0.074 | -0.14 (-2.69 to 2.41) | 0.917 |
| 48 | -2.05 (-3.81 to -0.29) | 0.022 | -2.63 (-4.83 to -0.44) | 0.019 | 0.49 (-2.30 to 3.27) | 0.731 |
| **SF_12 Physical (0-100)** |  |  |  |  |  |  |
| 24 | 4.99 (3.03 to 6.95) | <0.001 | 0.34 (-1.51 to 2.19) | 0.718 | 4.53 (1.86 to 7.20) | <0.001 |
| 48 | 3.75 (1.65 to 5.86) | <0.001 | 0.31 (-1.85 to 2.46) | 0.782 | 3.34 (0.34 to 6.35) | 0.029 |
| **PASE (0-400)** |  |  |  |  |  |  |
| 24 | 5.95 (-10.18 to 22.08) | 0.470 | 1.08 (-14.45 to 16.61) | 0.891 | 3.79 (-18.63 to 26.21) | 0.741 |
| 48 | -9.21 (-26.85 to 8.42) | 0.306 | -6.31 (-22.67 to 10.04) | 0.449 | -2.87 (-26.92 to 21.18) | 0.815 |
| **20-meter speed (m/s)** |  |  |  |  |  |  |
| 24 | 0.04 (0.00 to 0.07) | 0.039 | -0.01 (-0.04 to 0.03) | 0.706 | 0.04 (-0.01 to 0.09) | 0.106 |
| 48 | 0.01 (-0.03 to 0.05) | 0.650 | -0.03 (-0.07 to 0.00) | 0.079 | 0.04 (-0.01 to 0.10) | 0.143 |
| **The 5-time chair-to-stand tests (s)** |  |  |  |  |  |  |
| 24 | -1.50 (-2.23 to -0.77) | <0.001 | 0.15 (-0.42 to 0.73) | 0.607 | -1.61 (-2.54 to -0.67) | <0.001 |
| 48 | -1.28 (-2.17 to -0.38) | 0.005 | 0.18 (-0.52 to 0.87) | 0.620 | -1.43 (-2.54 to -0.31) | 0.012 |

Abbreviation: TKA, total knee arthroplasty; 95% CI = 95% confidence interval; WOMAC, western ontario and mcmaster university osteoarthritis; SF-12, the 12-item short form health Survey; PASE, the physical activity scale for the elderly.

Adjust for covariates at baselines: age, BMI, education, income, KL score of the right knee, Pain scores in the right knee, mental score of SF-12, the 5-time chair-to-stand tests (s).

Supplementary Table 2. Effect of TKA vs non-TKA after exclusion of participants in the TKA group (and matched controls) who underwent a contralateral TKA and follow-up data on participants in the non-TKA group from the implementation of a TKA

|  | **TKA** | |  | **non-TKA** | |  | **Between-Group Difference**  **TKA – non-TKA** | |
| --- | --- | --- | --- | --- | --- | --- | --- | --- |
|  | **beta (95% CI)** | **P** |  | **beta (95% CI)** | **P** |  | **beta (95% CI)** | **P** |
| **WOMAC Pain (0-20)** |  |  |  |  |  |  |  |  |
| 12 | -3.18 (-4.27 to -2.08) | <0.001 |  | -0.65 (-1.87 to 0.56) | 0.298 |  | -2.38 (-3.73 to -1.03) | <0.001 |
| 24 | -4.78 (-5.88 to -3.68) | <0.001 |  | 0.06 (-1.59 to 1.71) | 0.944 |  | -4.41 (-5.59 to -3.24) | <0.001 |
| 36 | -4.52 (-5.90 to -3.14) | <0.001 |  | 0.44 (-1.56 to 2.43) | 0.674 |  | -4.36 (-5.61 to -3.11) | <0.001 |
| 48 | -4.50 (-6.02 to -2.99) | <0.001 |  | 1.15 (-1.52 to 3.82) | 0.413 |  | -4.77 (-6.06 to -3.47) | <0.001 |
| 60 | -4.08 (-5.73 to -2.43) | <0.001 |  | 1.51 (-2.69 to 5.71) | 0.497 |  | -4.01 (-5.36 to -2.66) | <0.001 |
| **WOMAC Function (0-68)** |  |  |  |  |  |  |  |  |
| 12 | -10.66 (-14.14 to -7.18) | <0.001 |  | -2.03 (-6.16 to 2.11) | 0.345 |  | -7.84 (-11.97 to -3.70) | <0.001 |
| 24 | -14.97 (-18.22 to -11.71) | <0.001 |  | 0.45 (-4.90 to 5.80) | 0.870 |  | -13.59 (-17.52 to -9.66) | <0.001 |
| 36 | -14.36 (-18.44 to -10.28) | <0.001 |  | 1.59 (-4.68 to 7.86) | 0.625 |  | -13.57 (-17.70 to -9.43) | <0.001 |
| 48 | -14.79 (-19.39 to -10.19) | <0.001 |  | 2.51 (-6.24 to 11.26) | 0.584 |  | -14.69 (-19.21 to -10.17) | <0.001 |
| 60 | -13.27 (-18.59 to -7.94) | <0.001 |  | 3.35 (-8.76 to 15.46) | 0.600 |  | -12.40 (-16.69 to -8.11) | <0.001 |
| **SF-12 Mental (0-100)** |  |  |  |  |  |  |  |  |
| 24 | -2.37 (-5.59 to 0.85) | 0.165 |  | -1.05 (-6.51 to 4.41) | 0.713 |  | 0.04 (-2.47 to 2.54) | 0.976 |
| 48 | -2.57 (-6.42 to 1.28) | 0.211 |  | -3.43 (-10.99 to 4.13) | 0.396 |  | 0.73 (-1.92 to 3.38) | 0.589 |
| **SF_12 Physical (0-100)** |  |  |  |  |  |  |  |  |
| 24 | 5.30 (1.53 to 9.08) | 0.013 |  | -0.07 (-7.40 to 7.26) | 0.985 |  | 4.18 (1.45 to 6.91) | 0.003 |
| 48 | 4.46 (-0.06 to 8.98) | 0.073 |  | -1.11 (-12.47 to 10.26) | 0.855 |  | 2.90 (-0.07 to 5.87) | 0.057 |
| **PASE (0-400)** |  |  |  |  |  |  |  |  |
| 24 | 15.46 (-14.62 to 45.55) | 0.325 |  | 10.01 (-58.05 to 78.06) | 0.781 |  | 5.72 (-15.46 to 26.90) | 0.597 |
| 48 | 19.08 (-29.01 to 67.17) | 0.453 |  | -0.21 (-75.78 to 75.36) | 0.996 |  | -1.73 (-24.13 to 20.66) | 0.880 |
| **20-meter speed (m/s)** |  |  |  |  |  |  |  |  |
| 24 | 0.03 (-0.11 to 0.17) | 0.677 |  | 0.00 (-0.31 to 0.31) | 0.995 |  | 0.04 (-0.01 to 0.09) | 0.093 |
| 48 | -0.01 (-0.28 to 0.26) | 0.970 |  | -0.03 (-0.31 to 0.26) | 0.865 |  | 0.04 (-0.01 to 0.09) | 0.114 |
| **The 5-time chair-to-stand tests (s)** |  |  |  |  |  |  |  |  |
| 24 | -0.91 (-4.46 to 2.64) | 0.629 |  | -0.18 (-4.02 to 3.67) | 0.932 |  | -1.41 (-2.34 to -0.48) | 0.003 |
| 48 | -0.80 (-6.48 to 4.88) | 0.791 |  | -0.13 (-3.77 to 3.52) | 0.947 |  | -1.23 (-2.43 to -0.03) | 0.048 |

Abbreviation: TKA, total knee arthroplasty; 95% CI = 95% confidence interval; WOMAC, western ontario and mcmaster university osteoarthritis; SF-12, the 12-item short form health Survey; PASE, the physical activity scale for the elderly.

Adjust for covariates at baselines: age, BMI, education, income, KL score of the right knee, Pain scores in the right knee, mental score of SF-12, the 5-time chair-to-stand tests (s).
